# The Role of Adipokines as Circulating Biomarkers in Critical Illness and Sepsis

**DOI:** 10.3390/ijms20194820

**Published:** 2019-09-28

**Authors:** Sven H. Loosen, Alexander Koch, Frank Tacke, Christoph Roderburg, Tom Luedde

**Affiliations:** 1 Department of Medicine III, University Hospital RWTH Aachen, Pauwelsstrasse 30, 52074 Aachen, Germanyakoch@ukaachen.de (A.K.); tluedde@ukaachen.de (T.L.); 2 Department of Hepatology and Gastroenterology, Charité University Medicine Berlin, Augustenburger Platz 1, 10117 Berlin, Germany; Frank.tacke@charite.de; 3 Division of Gastroenterology, Hepatology and Hepatobiliary Oncology, University Hospital RWTH Aachen, Pauwelsstrasse 30, 52074 Aachen, Germany

**Keywords:** critical illness, sepsis, adipokines, biomarker, prognosis, ICU

## Abstract

Sepsis represents a major global health burden. Early diagnosis of sepsis as well as guiding early therapeutic decisions in septic patients still represent major clinical challenges. In this context, a whole plethora of different clinical and serum-based markers have been tested regarding their potential for early detection of sepsis and their ability to stratify patients according to their probability to survive critical illness and sepsis. Adipokines represent a fast-growing class of proteins that have gained an increasing interest with respect to their potential to modulate immune responses in inflammatory and infectious diseases. We review current knowledge on the role of different adipokines in diagnostic work-up and risk stratification of sepsis as well as critical illness. We discuss recent data from animal models as well as from clinical studies and finally highlight the limitations of these analyses that currently prevent the use of adipokines as biomarkers in daily practice.

## 1. Introduction

### 1.1. Critical Illness and Sepsis

Sepsis has recently been defined as “life-threatening organ dysfunction caused by a dysregulated host response to infection” [1,2,3]. In the United States, up to two percent of patients admitted to hospital suffer from sepsis. Half of these patients need treatment in an intensive care unit (ICU), representing 10% of all admissions [4]. Sepsis is still the leading cause of death among ICU patients. Between 28.3% and 41% of all sepsis patients will not survive their acute illness, with multi-organ failure being the most important cause of death [5]. In addition, survivors often show severe prolonged physical, neurological, and psychological limitations. Notably, the severity of sepsis correlates with the extent of post-sepsis disabilities in surviving patients [6], highlighting the need for early diagnosis and early treatment of this disease.

### 1.2. Biomarkers for Critical Illness and Sepsis

Adapting the diagnostic and therapeutic management to the personalized characteristics and needs of each individual patient represents one of the main challenges of precision medicine in the 21st century. In the last decade, many different treatment approaches were approved based on specific genetic characteristics such as anti-EGFR directed therapies in patients with *RAS wild-type* colorectal cancer [7]. Thus, providing individualized or personalized medicine requires the availability of specific “biomarkers” that allow stratification of patients into different subgroups [8]. The National Institutes of Health (NIH) define a biomarker as “any substance, structure, or process that can be measured in the body or its products and influence or predict the incidence or outcome of disease” or, more broadly, as “almost any measurement reflecting an interaction between a biological system and a potential hazard, which may be chemical, physical, or biological. The measured response may be functional and physiological, biochemical at the cellular level, or a molecular interaction” (reviewed in the work of [9]). Because, in recent years, manifold specific sepsis therapies have failed, current effective treatment strategies consist of detecting sepsis as early as possible, fluid resuscitation, and anti-infective treatments [1]. Especially easily accessible biomarkers, for example, from patients’ serum, might provide an important tool in the diagnostic process of sepsis at an early stage, to detect subgroups of patients with a high-risk profile and to monitor disease progression [10]. At present, microbiological cultures are widely accepted as the gold standard for diagnosis of septic disease [11]. However, in most cases, the results from microbiological cultures are only available days after sample collection and are associated with high rates of false negative results, leading to an ongoing search for other markers that might serve as surrogate for the presence of sepsis [12]. The C-reactive protein (CRP) has been identified as an inexpensive, but sensitive surrogate for infection and inflammation [13]. Named for its ability to bind the C-polysaccharide of *Streptococcus pneumoniae*, it represents an acute-phase protein that is secreted by hepatocytes in response to infection, inflammation, and tissue damage [13]. However, to distinguish between sepsis and non-infectious disease etiology, CRP lacks specificity, and elevated CRP serum levels are also common in patients with inflammatory (but not infectious) diseases states such as cancer, thromboembolic- or cardiovascular diseases, and burns [13]. Moreover, owing to the hepatic provenience of CRP, in the case of severe liver failure, CRP levels may be falsely low and may rather reflect the degree of hepatic dysfunction than sepsis or inflammation [14], limiting its use in clinical routine. Besides CRP, procalcitonin (PCT) was more recently established in clinical routine to identify patients with septic disease. PCT represents a precursor-hormone of calcitonin. It is produced by various organs and, in healthy individuals, the liver is considered to be the most important source of PCT [15]. During infection, PCT is secreted by cytokine-activated macrophages as well as by the parenchyma of many organs, leading to a dramatic increase in circulating PCT levels [15]. Despite that different guidelines recommend the use of PCT to distinguish between sepsis and non-septic cause of disease in critically ill patients, recent meta-analyses have demonstrated that the use of PCT for guidance of therapy had no influence on further therapeutic procedures for ICU patients [11,16]. Moreover, the use of PCT is further hampered by the lack of a clear cut-off defining sepsis in patients with critical illness [17].

In addition to CRP and PCT, interleukin-6 (IL-6) has been suggested as a biomarker in the context of critical illness and sepsis. IL-6 represent the most potent inducer for the secretion of acute phase proteins in the liver. IL-6 serum concentrations were elevated in the blood of patients with septic disease and IL-6 concentrations were associated with the severity of sepsis. Moreover, patients with elevated IL-6 levels demonstrated an impaired prognosis compared with patients with lower levels [18]. However, similar to CRP and PCT, IL-6 concentrations were also elevated in non-septic disease states, limiting its specificity for the assessment of sepsis in patients with critical illness [19]. Therefore, owing to a lack in specificity and sensitivity of available markers for critical illness and sepsis, innovative biomarkers reflecting novel pathophysiological concepts are eagerly awaited to improve the outcome of patients treated on medical ICU. In this context, adipokines might represent biological plausible markers for diagnosis of sepsis and prognosis estimation in critically ill patients, because many processes that are regulated by or reflected by adipokines are involved in the pathophysiology of critical illness and sepsis. As an example, hyperglycemia, impaired glucose tolerance, and insulin resistance are commonly observed in critically ill patients with sepsis or septic shock and have been correlated with an impaired prognosis of these patients [20]. On the basis of these recent findings, many authors have measured serum concentrations of different adipokines in serum and plasma of critically ill patients with or without sepsis. Here, we review these previous data on the role of adipokines in diagnostic work-up and risk stratification of sepsis as well as critical illness. 

## 2. Adipokines

Until recently, the adipose tissue was only considered an energy storage organ, but many novel studies have demonstrated that it is deeply integrated into different physiological and pathophysiological regulatory processes including the regulation of diseases associated with systemic inflammatory responses [21]. In this context, it became obvious that the adipose tissue is able to secrete different factors that are collectively referred to as adipokines [22]. In obese patients, the secretory profiles of adipose tissue are decisively altered dependent on the degree of overweight. Secretion of almost all known adipokines is upregulated in patients with obesity, promoting systemic inflammation and the development of metabolic diseases. In detail, next to leptin, TNF, and IL-6, elevated levels of a broad panel of other proinflammatory adipokines (resistin, retinol-binding protein 4 (RBP4), lipocalin 2, IL-18, angiopoietin-like protein 2 (ANGPTL2), monocyte chemoattractant protein 1 (MCP1), CXC-chemokine ligand 5 (CXCL5), nicotinamide phosphoribosyltransferase (NAmPT)) were found to be upregulated in obese patients (reviewed in the work of [21]). In contrast, adiponectin expression was shown to be lower in adipocytes of obese patients. In line, data from animal models revealed that adiponectin is protective against metabolic and cardiovascular diseases that might develop in the context of obesity. Thus, it seems likely that the inflammatory response and metabolic dysfunction occurring in patients with obesity represent the consequence of a disturbed balance in the secretion of pro- and anti-inflammatory adipokines.

The concept of adipokines as regulators of body homeostasis and responders to injurious threats might be of tremendous relevance for understanding the pathophysiology of critical illness and sepsis. Moreover, concentrations of adipokines can be easily determined in clinical routine, and different authors have suggested using adipokines as biomarkers in treated ICU patients. Within this review, we aim at summarizing available data on the most relevant adipokines in critical illness and sepsis and discuss limitations of the present analyses that have prevented the use of adipokine measurements in clinical routine until now.

## 3. Selected Adipokines with a Potential Role in Critical Illness and Sepsis

The following section will give an overview of the most relevant adipokines in the context of critical illness as sepsis. Table 1 summarizes the most significant findings with respect to adipokine regulation in critically ill patients.

### 3.1. Omentin

Omentin represents a relatively new member of the adipokine family and is mainly secreted by the visceral adipose tissue [23]. Experimental data support a decisive role of omentin in the inflammatory crosstalk. As such, it negatively influences a TNFa dependent activation of well-known inflammatory signaling pathways such as p38 or JNK. Clinical data further suggest aberrant omentin serum levels in patients with obesity, inflammatory bowel disease, diabetes mellitus, or coronary heart disease [24,25].

In patients with critical illness and/or sepsis, only little is known on a potential role of omentin as a biomarker for the assessment of disease severity or the patients’ clinical outcome. Our group has assessed omentin serum levels in a cohort of *n* = 117 ICU patients with different disease etiologies. While serum omentin levels at admission to the ICU or 72 h after did not differ between ICU patients and healthy controls and were independent of disease etiology and the presence of sepsis, low omentin serum levels were an independent predictor for overall survival [26]. However, these data need further validation before omentin might be implemented into clinical risk prediction scores.

### 3.2. C1q/TNF-Related Protein 1 (CTRP1)

The CTRP1 represents a member of the CTRP family that consists of 15 proteins that are involved in numerous physiological and pathophysiological processes such as the immune defense, systemic inflammation, cell differentiation and apoptosis, and autoimmunity [27]. CTRP1 is known to regulate important processes within the systemic energy homeostasis and insulin sensitivity and is, for example, involved in PI3K-dependent signaling pathways to induce intracellular glucose transport by insulin [28,29]. Moreover, CTRP1 stimulates human vascular smooth muscle cells, which results in an upregulation of pro-inflammatory cytokines such as interleukin 6 (IL-6), monocyte chemoattractant protein 1 (MCP1), and intracellular adhesion molecule 1 (ICAM1) [30].

In critically ill patients, recent data suggest a significant upregulation of CTRP1 compared with healthy controls [31]. Moreover, CTRP1 levels were significantly higher in patients who fulfilled the criteria of sepsis compared with non-sepsis patients. Importantly, our group showed a strong correlation with markers of systemic inflammation (CRP, IL-6, and PCT), obesity/diabetes (BMI and HbA1c), liver function and cholestasis (bilirubin, GGT, GLDH, AP), and renal function (creatinine, urea). Although these data clearly link CTRP1 to inflammatory and metabolic disturbance in critically ill patients, especially in those patients with septic disease stage, circulating CTRP1 levels were not indicative for the patients’ short- or long-term mortality [31]. In a different cohort of *n* = 539 patients undergoing coronary angiography for the evaluation of coronary artery disease, CTRP1 was likewise correlated to obesity and the presence of metabolic syndrome or type 2 diabetes, but circulating levels of CTRP were also a significant predictor of major adverse cardiovascular events such as cardiovascular death, non-fatal myocardial infarction, and non-fatal stroke over a follow-up period of eight years [32].

### 3.3. C1q/TNFa-Related Protein 3 (CTRP3)

Similar to CTRP1, CTRP3 is a recently recognized adipokine that is also involved in various physiological and pathophysiological processes such as food intake and metabolism, inflammation, vascular disorders, and tumor metastases [33]. CTRP3 is expressed by visceral and subcutaneous adipose tissue [34] and is generally referred to as a “beneficial” mediator as it lowers levels of glucose, inhibits gluconeogenesis, and exerts anti-inflammatory effects [35,36]. In line, serum levels of CTRP3 are reduced in patients with obesity or diabetes [29].

In terms of septic disease, animal models suggest an anti-inflammatory and protective role of CTRP3. As an example, intramyocardial overexpression of CTRP3 in mice resulted in a significantly attenuated myocardial dysfunction in an LPS-induced model of sepsis [37]. In human critical illness, circulating CTRP3 levels were found to be reduced. In a cohort of *n* = 218 patients, both critically ill patients with (*n* = 145) or without (*n* = 73) sepsis had significantly decreased plasma CTRP3 levels compared with healthy controls [38]. Moreover, low CTRP3 levels were directly associated with the presence of sepsis among ICU patients. Interestingly, we did not observe an association of CTRP3 levels with obesity or diabetes in ICU patients, arguing that critical illness might overrule the regulation mechanisms of CTRP3 in non-critically ill patients. In line, CTRP3 plasma concentrations were inversely correlated with inflammatory cytokines and standard markers of sepsis such as CRP and PCT. Importantly, the authors described a direct association between low CTRP3 levels (<620.6 ng/mL) and an increased overall mortality [38]. Although these data need to be confirmed in further trials, it suggests a clinically relevant role of CTRP3 as a potential new diagnostic and prognostic biomarker in patients with critical illness and sepsis.

### 3.4. Leptin and Leptin-Receptor

Leptin represents a 16 kDa hormone that was initially described in 1994 and has since been extensively studied for its regulatory role with respect to food intake, glucose homeostasis, and energy expenditure [39,40]. It mainly originates from adipocytes and was also found to be involved in cell-mediated immunity as well as inflammatory cytokine crosstalk [41]. There is a direct correlation of circulating leptin levels with body fat mass; starvation or malnutrition leads to low serum concentrations, while obesity increases leptin serum levels [42].

Several clinical studies have evaluated the role of circulating leptin as a biomarker in the context of critical illness, but the results are partly inconclusive. In a first study of *n* = 137 critically ill patients (95 with sepsis, 42 without sepsis), serum leptin concentrations at admission to ICU were similar compared with healthy controls and did not differ between septic and non-septic patients [43]. Similar results were obtained for the soluble leptin receptor, which is known to form complexes with circulating leptin. In line, a smaller series of patients with severe sepsis revealed no significant regulation of serum leptin levels in septic disease [44]. Contrarily, serum leptin levels were significantly elevated in septic patients compared with non-septic patients in a cohort of *n* = 331 patients with critical illness from a medical ICU [41]. In a longitudinal study including patients with sepsis, leptin levels were significantly higher compared with controls and showed a positive correlation with insulin levels and insulin resistance. Interestingly, a decline of leptin serum levels was found during prolonged sepsis, which was, however, not related to survival [45]. A potential explanation for these contradictory results might relate to different sampling time points, divergent BMI or feeding state prior and during sepsis, or from heterogeneous cohorts in terms of disease etiology.

In terms of a prognostic marker, low leptin serum levels (<10 ng/mL) were associated with an adverse outcome in a cohort of 230 adult patients with severe secondary peritonitis [46]. Moreover, elevated leptin-receptor receptor levels (>32 ng/mL) were associated with an impaired overall survival compared with patients with low serum levels (<32 ng/mL) [44].

### 3.5. Visfatin

Visfatin, which is also referred to as pre-B-cell colony-enhancing factor (PBEF) given its initial identification in lymphocytes, represents an adipokine with key functions in the process of systemic inflammation. It was shown that visfatin acts as a chemoattractant to recruit neutrophils and promotes their survival [47,48], and is further capable of stimulating cytokine release from monocytes [49]. Visfatin has also been associated with human critical illness and sepsis and was primarily suggested as a diagnostic marker. In septic infants, visfatin serum levels were significantly elevated compared with healthy controls and showed a positive correlation with CRP, PCT, and IL-6 [50]. At a cut-off value of 10 ng/mL, visfatin revealed a sensitivity and specificity of 92% and 94%, respectively, for the diagnosis of neonatal sepsis.

In a larger study including 229 critically ill medical ICU patients, circulating visfatin levels were also significantly higher in ICU patients when compared with healthy controls [51]. Visfatin serum levels were highest in patients who fulfilled the diagnostic criteria for sepsis, and visfatin concentrations strongly correlated with disease severity and organ failure. Although visfatin serum levels were comparable between patients with or without type 2 diabetes or obesity, the authors observed a significant correlation between visfatin levels and biomarkers of liver and kidney dysfunction and other adipokines such as resistin and leptin. Most importantly, high visfatin levels upon admission to ICU were associated with both an increased ICU as well as long-term mortality [51].

### 3.6. Resistin

Resistin was first described in 2001 as an adipokine. Data from rodent models revealed an association between resistin and the presence of metabolic diseases including obesity and type 2 diabetes. In these studies, elevated serum levels of glucose as well as hyperinsulinemia correlated with an increase in resistin [52]. When translating data from animal models to humans, it is important to consider that the protein sequences of murine and human resistin demonstrate important differences [53]. Moreover, in mice, adipocytes represent the main source of circulating resistin, while in humans, resistin seems to be mainly secreted by macrophages [54,55], suggesting that the role of resistin in humans and mice may vary. In this context, data from a ‘humanized mouse’ model in which only human resistin was produced by macrophages suggested that human resistin might display pro-inflammatory characteristics mediating insulin resistance [56]. Therefore, a role of resistin in the pathophysiology of critical illness and sepsis seemed likely. Just recently, it was demonstrated that serum levels of resistin are elevated in patients with critical illness compared with controls [20]. Moreover, patients with sepsis displayed further elevated resistin concentrations compared with non-septic patients, suggesting a link between inflammation and infection and the secretion of resistin in humans. In line, serum resistin concentrations were closely correlated to inflammatory parameters such as CRP, the leukocyte count, PCT, and cytokines such as IL6 and TNF [20]. Moreover, elevated resistin levels indicated an impaired prognosis [20]. Notably, these data were subsequently corroborated by similar findings of different groups, demonstrating that elevated resistin concentrations correlate with disease severity, inflammatory cytokine, lactate levels, and serum creatinine concentrations in patients with severe sepsis and septic shock [57,58]. These data might at least partly be explained by the fact that resistin represents a uremic toxin, inhibiting neutrophils and thereby modulating sepsis-related immune responses at concentrations that can be found in patients with end-stage kidney failure [59]. In line with these data from adult patients, recently, Gokmen et al. demonstrated that resistin levels are elevated in preterm infants with sepsis, concluding that resistin may represent a marker for sepsis in premature infants [60].

### 3.7. Adiponectin

Adiponectin represents an adipokine of 30 kDa that is exclusively secreted by adipocytes and has been extensively studied for its role in glucose and lipid metabolism and in the context of insulin resistance [61]. In obese and patients with diabetes mellitus, circulating levels of adiponectin are reduced compared with healthy individuals [62]. Moreover, circulating adiponectin levels negatively correlate with serum levels of low-density lipoprotein cholesterol and triglycerides, blood pressure, and insulin resistance [63], arguing for a protective function of adiponectin in human metabolic homoeostasis. This concept is supported by animal studies suggesting an anti-inflammatory and protective role of adiponectin in mouse models of sepsis [64,65].

Only limited data on a regulation of adiponectin in critical illness and sepsis are available. In a study evaluating circulating levels of adiponectin in 170 critically ill patients at admission to the ICU, the authors found comparable adiponectin levels in ICU patients with or without sepsis and healthy controls [66]. Similar to patients without critical illness, ICU patients with obesity or preexisting diabetes mellitus displayed significantly reduced levels of circulating adiponectin. Interestingly, although not regulated in ICU patients, the authors found that low adiponectin levels at ICU admission are an independent positive predictive marker for short-term and overall survival [66]. During the clinical course of critical illness, two studies have reported low to normal levels of circulating adiponectin. In a small series of patients, plasma adiponectin concentrations in an ICU cohort at day 3 and day 7 after admission were significantly lower compared with healthy control samples [67]. In line, a study including 318 patients with respiratory critical illness revealed low adiponectin levels, which were enhanced by insulin therapy and returned to normal when critical illness was sustained [68].

### 3.8. Retinol Binding Protein 4

Retinol binding protein 4 (RBP4) is involved in the transport of hepatic retinol to distant organs. Levels of circulating RBP4 have been linked to metabolic diseases such as insulin resistance, obesity, metabolic syndrome, diabetes, and fatty liver disease, representing chronic inflammatory diseases [68]. Recently, the group of Langouche et al. demonstrated in 318 critically ill patients that those patients with sepsis had significantly lower RBP4 levels than those with other disease etiologies [68]. In a different study, RBP4 levels were also lower in ICU patients, regardless of whether or not sepsis was present, compared with controls [69]. Interestingly, in these patients, liver cirrhosis was associated with further reduced RBP4 concentrations and RBP4 was correlated with markers of liver dysfunction. Interestingly, RBP4 concentrations were independent on the presence of obesity or preexisting diabetes and were not associated with overall survival in the analyzed cohort. In contrast, Chen et al. demonstrated that baseline RBP levels predicted short-term mortality in critically ill patients with underlying liver disease. Interestingly, in this analysis, those patients that survived ICU treatment displayed significantly increased RBP4 levels after ICU discharge [70], highlighting the potential of this biomarker in predicting survival in patients treated on a medical ICU.

## 4. Discussion and Outlook

Adipokines represent a growing class of proteins that exert a wide range of metabolic effects. Moreover, adipokines have been shown to withhold regulatory effects in immune responses during inflammation and infectious disease. Because adipokines are secreted into the blood, recent studies have analyzed their potential as biomarkers in many different pathological conditions. While many papers found an association between serum levels of adipokines and the presence of sepsis and/or the prognosis of patients with critical illness, it is important to highlight some limitations that apply at least for most of the available studies. Many of the available studies featured a retrospective design and the missing longitudinal approach may have induced a bias. Moreover, some of the analyzed cohorts were extremely heterogeneous and relatively small in terms of patients’ numbers; therefore, larger prospective studies are needed to finally infer about the role of circulating adipokines in patients with critical illness, a collective of patients with a still unacceptably poor prognosis. Within this review, we summarized available data on the potential role of these proteins in critically ill and septic patients. We highlight that, despite that their use as single marker might be limited owing to a lack of sensitivity and specificity, measurements of circulating adipokines might be integrated into available and future scoring systems for the diagnosis of sepsis and risk stratification of critically ill patients.

## Figures and Tables

**Table 1:** Overview of regulated adipokines in critically illness and sepsis.

	Adipokine	Circulating Adipokine Levels in Critical Illness and/or Sepsis	Prognostic Relevance?	Reference
1	Omentin	−	+	[26]
2	CTRP1	↑	−/(+)	[31,32]
3	CTRP3	↓	+	[38]
4	Leptin and Leptin Receptor	−/↑	+	[41,43,44,45,46]
5	Visfatin	↑	+	[50,51]
6	Resistin	↑	+	[20,57,58]
7	Adiponectin	−/↓	+	[66,67,68]
8	RBP4	↓	−/(+)	[68,69,70]

CTRP1: C1q/TNFa-related protein 1, CTRP3: C1q/TNFa-related protein 3, RBP4: retinol binding protein 4, −: no regulation/no prognostic relevance, +: prognostic relevance, ↑: elevated circulating levels, ↓: decreased circulating levels.
